# Synergistic Activity of Eugenol, Cinnamaldehyde, and Carvacrol in Combination with Different Antibacterial Agents Against Multidrug-Resistant Gram-Negative Clinical Isolates

**DOI:** 10.3390/antibiotics15040391

**Published:** 2026-04-11

**Authors:** Rocco Latorre, Maria Chiara Valerii, Irene Ferrari, Marco Benati, Enzo Spisni, Alessia Pardo, Massimo Albanese, Caterina Signoretto, Giuseppe Lippi, Paolo Gaibani

**Affiliations:** 1 NYU Pain Research Center, New York University, New York, NY 10010, USA; rl3423@nyu.edu; 2 Department of Molecular Pathobiology, College of Dentistry, New York University, New York, NY 10010, USA; 3 Department of Biological, Geological and Environmental Sciences, University of Bologna, 40126 Bologna, Italy; mariachiara.valerii2@unibo.it (M.C.V.); enzo.spisni@unibo.it (E.S.); 4 Microbiology Section, Department of Diagnostic and Public Health, University of Verona, 37134 Verona, Italy; irene.ferrari_05@studenti.univr.it (I.F.); caterina.signoretto@univr.it (C.S.); 5 Department of Pathology, Azienda Ospedaliera Universitaria Integrata di Verona, 37134 Verona, Italy; marco.benati@univr.it (M.B.); giuseppe.lippi@univr.it (G.L.); 6 Section of Clinical Biochemistry, Department of Engineering for Innovative Medicine (DIMI), University of Verona, Piazzale L.A. Scuro 10, 37134 Verona, Italy; 7 Maxillofacial Surgery and Dentistry Section, Department of Surgery, Dentistry, Pediatrics and Gynecology, University of Verona, 37124 Verona, Italy; alessia.pardo@univr.it (A.P.); massimo.albanese@univr.it (M.A.); 8 Microbiology and Virology Unit, Azienda Ospedaliera Universitaria Integrata di Verona, 37134 Verona, Italy

**Keywords:** essential oils, KPC-producing *K. pneumoniae*, CRAB, colistin, synergy

## Abstract

**Background/Objectives**: The WHO has identified carbapenem-resistant *Acinetobacter baumannii* (CRAb) and carbapenem-producing Enterobacterales (CPE) as the “critical priority” group of multidrug-resistant (MDR) organisms for which new therapeutic strategies are urgently needed. Here, we evaluated the in vitro synergistic activity of eugenol, cinnamaldehyde, and carvacrol in combination with β-lactams, gentamicin, or colistin against MDR Gram-negative bacteria (GNB). **Methods**: We selected seven MDR-GNB clinical isolates including CRAb, ESBL-producing and CPE clinical isolates displaying different antimicrobial susceptibility profiles. The genomes of clinical isolates were characterized by whole-genome sequencing and synergy testing was performed with checkerboard assay. **Results**: Our results demonstrate that eugenol, cinnamaldehyde, and carvacrol in combination with colistin exhibited synergistic activity (FICI < 0.5) against MDR-GNB clinical isolates ranging from 37.5 to 50%, while the effect was almost indifferent in combination with different β-lactam molecules or gentamicin against 87.5–100% of MDR-GNB strains. The synergistic interaction of eugenol, cinnamaldehyde, and carvacrol with colistin induced a statistically significant reduction (*p* < 0.05) in the MIC values compared with the molecules tested alone. **Conclusions**: Our data demonstrate that this synergistic interaction was not affected by different antimicrobial resistance genes and/or different antimicrobial susceptibility profiles. In conclusion, our results suggest that eugenol, cinnamaldehyde, and carvacrol in combination with colistin represent a potential strategy for the treatment of MDR-GNB pathogens and limit their diffusion.

## 1. Introduction

Antimicrobial resistance (AMR) has transitioned from an emerging biological concern to one of the most urgent global health threats, undermining decades of effective infection management and jeopardizing essential medical procedures such as major surgery, transplantation, and chemotherapy [1,2]. Recent global estimates suggest that AMR is directly responsible for over one million deaths each year, a burden that is projected to worsen without coordinated international action [2]. Among resistant pathogens, Gram-negative bacteria (GNB) represent the most formidable challenge due to their inherently low membrane permeability, active efflux systems, and remarkable capacity to acquire and disseminate mobile genetic elements encoding resistance determinants [3]. These characteristics not only facilitate multidrug resistance (MDR) but also limit the efficacy of many newly developed antibiotics.

To rationalize global R&D and public health priorities, the World Health Organization (WHO) developed the Bacterial Priority Pathogens List (BPPL) in 2017 and revised it in 2024, incorporating updated epidemiological, therapeutic, and clinical criteria to identify organisms posing the greatest threat to human health [4,5]. The 2024 BPPL assigns 24 pathogens into critical, high, and medium priority groups, integrating parameters such as disease burden, transmission potential, preventability, and availability of effective treatments [4]. Within the critical priority tier, three groups of GNB stand out due to their widespread distribution, increasing resistance rates, and limited therapeutic options: carbapenem-resistant *Acinetobacter baumannii* (CRAB), carbapenem-resistant Enterobacterales (CRE), and third-generation cephalosporin-resistant Enterobacterales (3GCRE) [4,5]. These organisms are associated with high mortality rates, particularly in hospitalized and critically ill populations, representing significant drivers of morbidity, prolonged hospitalization, and healthcare costs [1,2].

CRAB has become emblematic of the challenges posed by MDR-GNBpathogens, especially in intensive care units, where the immunological status of recovered patients contributes to creating a favorable environment for ventilator-associated pneumonia, bloodstream infections, wound infections, and urinary tract infections [3]. CRAB isolates often display resistance to nearly all antibiotic classes due to a complex repertoire of mechanisms. Carbapenem resistance is primarily mediated by OXA-type carbapenemases, including OXA-23, OXA-24/40, OXA-58, and the intrinsic OXA-51 family, while the emergence of metallo-β-lactamases such as NDM further complicates treatment [3]. Additional mechanisms include the downregulation or loss of porins, the overexpression of resistance–nodulation–division efflux pumps, and alterations in penicillin-binding proteins, all of which contribute to extensive drug resistance [3]. As a result, CRAB infections often leave clinicians with extremely limited therapeutic choices and are responsible for mortality rates exceeding 40% in some settings [1,3].

Enterobacterales represent a major global AMR burden [1,2]. The spread of carbapenemase-producing Enterobacterales (CPE), especially strains producing KPC, NDM, VIM, IMP, or OXA-48-like enzymes, has drastically reduced the effectiveness of carbapenems against ESBL- or AmpC-producing organisms [5]. Some metallo-β-lactamases, particularly NDM, confer resistance to nearly all β-lactams except aztreonam, while the co-production of ESBLs or AmpC enzymes often renders aztreonam ineffective unless combined with novel β-lactamase inhibitors [6]. Meanwhile, third-generation cephalosporin-resistant Enterobacterales (3GCRE) frequently harbor CTX-M-type ESBLs or plasmid-mediated AmpC enzymes that compromise the effectiveness of cephalosporins, fluoroquinolones, and aminoglycosides [1,4]. These organisms are implicated in a wide range of clinical syndromes, including urinary and intra-abdominal infections, pneumonia, and even sepsis.

Therapeutic options for the WHO critical priority pathogens remain exceedingly limited [4]. Polymyxins such as colistin continue to be widely used for CRAB and CRE infections, although their clinical utility is constrained by nephrotoxicity, neurotoxicity, suboptimal pharmacokinetics, and growing resistance driven by chromosomal mutations and plasmid-borne *mcr* genes [7]. Alternative agents for CRAB, such as tigecycline and minocycline, display variable susceptibility patterns and may not achieve adequate serum concentrations for bloodstream infections [3]. Newer β-lactam/β-lactamase inhibitor combinations, including ceftazidime-avibactam, meropenem-vaborbactam, and imipenem-relebactam, have improved the management of KPC-producing Enterobacterales but lack activity against metallo-β-lactamases, especially NDM [6]. Cefiderocol, a siderophore cephalosporin with activity against many CRE and CRAB isolates, showed initial promise, however emerging resistance mechanisms, such as mutations affecting siderophore uptake pathways or β-lactamase overexpression, have been increasingly reported [8]. Collectively, these observations underscore the need for novel antimicrobial approaches that can restore antibiotic activity, circumvent established resistance mechanisms, or function synergistically with existing agents.

In recent years, considerable interest has turned toward natural antimicrobial compounds, especially components of essential oils (EOs), which possess broad-spectrum antibacterial, anti-biofilm, and anti-virulence properties. EO constituents such as cinnamaldehyde, eugenol, and carvacrol have been widely studied for their diverse mechanisms of action, multi-target activity, and potential to synergize with conventional antibiotics [9,10]. The multifaceted nature of these molecules, ranging from membrane disruption and increased permeability to impairment of ATP production, inhibition of quorum sensing, and interference with bacterial metabolism, reduces the likelihood of developing resistance compared to single-target antibiotics.

Growing evidence indicates that EO components can potentiate the activity of β-lactams, aminoglycosides, and polymyxins against MDR bacteria. Cinnamaldehyde and eugenol have been demonstrated to reduce β-lactam resistance in *A. baumannii* by altering outer membrane stability, inhibiting efflux activity, and increasing antibiotic uptake [9]. Carvacrol has been shown to exert direct bactericidal activity via membrane perturbation, induction of oxidative stress, and ribosomal dysfunction, while also displaying synergistic behavior with antibiotics against Gram-positive and Gram-negative pathogens [10]. In Enterobacterales, synergistic interactions between EO components and antibiotics have been observed in both ESBL-producing and colistin-resistant *K. pneumoniae*, with reports demonstrating reduced MIC values, disruption of biofilm architecture, and inhibition of biofilm-associated resistance phenotypes [11]. Moreover, combinations of thymol or carvacrol with β-lactams or aminoglycosides have shown the capacity to enhance bacterial killing and to suppress the emergence of resistant subpopulations, offering a potential role in resistance prevention strategies [12].

However, significant gaps remain in the systematic evaluation of EO–antibiotic combinations specifically targeting CRAB, CRE, and 3GCRE, the organisms for which therapeutic innovation is most urgently needed according to the WHO BPPL [4]. Many existing studies focus on single compounds, limited bacterial species, or non-clinical isolates, and relatively few have systematically examined interactions with clinically important antibiotics such as “ceftazidime, cefepime, meropenem, colistin, or gentamicin” across panels of genome-characterized clinical isolates. Addressing these gaps is essential for clarifying whether EO components could be used as viable adjunctive agents for MDR-GNB infections.

Therefore, the present study aims to investigate the antibacterial activity and potential synergistic interactions of cinnamaldehyde, eugenol, and carvacrol in combination with selected clinically relevant antibiotics against MDR-GNB isolates, including CRAB and CRE strains. By evaluating their effects on antibiotic MICs, assessing their ability to restore susceptibility in resistant strains, and analyzing synergistic activity, this work seeks to explore evidence-based adjunctive strategies that may ultimately contribute to improved treatment options for these WHO-designated critical priority pathogens.

## 2. Materials and Methods

### 2.1. Phenotypic Analysis

Bacterial identification was performed by Matrix-Assisted Laser Desorption/Ionization Time-of-Flight (MALDI-TOF) mass spectrometry (MS) assay (Bruker Daltonics, Bremen, Germany). Preliminary antimicrobial susceptibility profiles were assessed using the MicroScan Walkaway system, and MICs were confirmed by Sensititre^TM^ Plate EUMDRXXF (Thermofisher, Waltham, MA, USA). Results were interpreted following the European Committee on Antimicrobial Susceptibility Testing (EUCAST) clinical breakpoints v15.0 [13].

Final MIC confirmation was performed by broth microdilution (BMD) following standardized protocols [14]. Briefly, serial two-fold dilutions of each cinnamaldehyde, eugenol, carvacrol, and antibiotics were prepared in Mueller–Hinton II broth across a concentration range of 0.06 to 4.096 mg/L. Bacterial suspensions were prepared from pure overnight cultures and adjusted to a 0.5 McFarland standard. Then, bacterial suspensions were diluted in Mueller–Hinton broth to a final concentration of 5 × 10^5^ CFU/mL. Microtiter plates were inoculated and incubated at 35 ± 2 °C for 18–24 h, and MIC was defined as the lowest concentration of antibiotic that completely inhibited visible bacterial growth. Each sample was tested in triplicate. Carbapenemase production was identified by the NG-Test CARBA 5 (NG Biotech, Guipry-Messac, France).

All MDR clinical strains were tested against cinnamaldehyde, eugenol, carvacrol, and against different antimicrobials, including cefepime, ceftazidime, meropenem, colistin, and gentamicin. The BL21 competent *E. coli* strain (Thermofisher, Waltham, MA, USA) was used as a control based on the well-characterized susceptibility pattern to different antimicrobials used in this study. MIC evaluation was performed by BMD as previously described [14]. Briefly, serial two-fold dilutions of each antibiotic were prepared in Mueller–Hinton broth across a concentration range of 0.032 to 4096 mg/L. Bacterial inocula were prepared in saline solution (NaCl 0.9%) and adjusted to a concentration of 0.5 McFarland standard, then diluted in Mueller–Hinton broth to a final concentration of 5 × 10^5^ CFU/mL. Microtiter plates were inoculated and incubated at 35 ± 2 °C for 18–24 h, and MIC was defined as the lowest concentration of antimicrobials that completely inhibited visible bacterial growth. Each sample was tested in triplicate.

### 2.2. Genomic Analysis

Whole-genome sequencing of clinical strains included in this study was performed as previously described [15]. Briefly, bacterial genomic DNA was extracted from bacterial cultures of *K. pneumoniae* and *A. baumannii* using DNeasy Blood&Tissue Kit (Qiagen, Basel, Switzerland) and cleaned up with AMPure XP magnetic beads (Beckman Coulter, Mumbai, Maharashtra). Libraries were prepared with the DNA Prep Library Preparation Kit (Illumina, San Diego, CA, USA) and sequenced by the Illumina MiSeq system (Illumina, San Diego, CA, USA) using MiSeq Reagent Kit v.3 with 2 × 300 paired-end reads. Prior to genome assembly, a filtering step was conducted to remove any contaminant human reads. Briefly, a read quality evaluation was performed with FastQC 0.11.9 (https://www.bioinformatics.babraham.ac.uk/projects/fastqc/, accessed on 1 January 2023) and aggregated MultiQC reporting before and after filtering. A minimum Q score of 30 was set to filter Illumina reads, and filtered sequencing reads were de novo assembled using SPAdes software v3.15.2. Antimicrobial resistance genes and MLST analysis were determined with the CGE tool (available at: https://genepi.food.dtu.dk/, accessed on 1 January 2025). Antimicrobial resistance genes were determined using the ResFinder web tool (https://genepi.food.dtu.dk/resfinder, last accessed on 1 January 2023). Porin genes were aligned against the respective amino acid sequence retrieved from the database of the National Center for Biotechnology Information (NCBI; *OmpK35* [WP_004141771.1] and *OmpK36* [WP_002913005.1] for *K. pneumoniae*; OprD [WP_000910004] and CarO [ABC46545.1] for *A. baumannii*) with the blastx command.

### 2.3. Cinnamaldehyde, Eugenol, Carvacrol, and Antibiotic Powder Preparation

All antibiotic stock solutions were dissolved in sterile water to the appropriate concentrations and stored at −80 °C. For cinnamaldehyde, eugenol, and carvacrol, powders were resuspended in water with the addition of dimethyl sulfoxide (DMSO) solution. To establish the optimal concentration of DMSO used for resuspension of cinnamaldehyde, eugenol, and carvacrol, different concentrations ranging from 2% to 50% were tested to establish the effect against bacterial cells, as previously described [16]. Briefly, exponentially growing cultures were diluted to a final concentration of 10^5^ CFU/mL and tested in the presence of DMSO at 2%, 5%, 10%, and 50% *v*/*v*. Viable colony counts were determined after an overnight incubation at 37 °C, and the percentage of viable cell rates was determined in comparison to control growth without DMSO addition.

### 2.4. Synergy Evaluation

Checkerboard assays were performed in a 96-well plate filled with 50 μL of Mueller–Hinton broth per well. Cinnamaldehyde, eugenol, and carvacrol were serially diluted along the vertical axis, while antibiotics were serially diluted along the horizontal axis. Inoculum was prepared to a 0.5 McFarland standard and diluted to 5 × 10^5^ CFU/mL, and each well was filled with 100 μL of bacterial suspension. Plates were incubated at 35–37 °C for 24 h under aerobic conditions. The MIC of each antibiotic in combination was recorded, and synergy was evaluated by calculating the Fractional Inhibitory Concentration (FIC) Index (FICI), as previously described by Carretto et al. [17]. Briefly, FICI was calculated as follows: FICI = FIC of agent A + FIC B, where FIC A is the MIC of the combination/MIC of drug A alone, and FIC B is the MIC of the combination/MIC of drug B alone. FICI results were interpreted as synergy, FICI ≤ 0.5; indifferent, 0.5 > FICI ≤ 4; antagonism, FICI ≥ 4 [18].

## 3. Results

### 3.1. Genomic Characteristics of MultiDrug-Resistant Gram-Negative Strains

Assembly produced draft genomes of 5,605,491 bp (LE08), 5,551,963 bp (NA28), 5,575,630 bp (Kp86), 5,819,432 bp (Kp61), 5.385,310 bp (Kp51), 4,011,238 bp (TO35), and 3,871,701 bp (TO28) composed, respectively, by 101 (LE08), 74 (NA28), 66 (Kp86), 90 (Kp61), 32 (Kp51), 142 (TO35), and 63 (TO28) contigs. The genomes have 57.16% (LE08), 57.12% (NA28), 57.22% (Kp86), 56.88% (Kp61), 57.29% (Kp51), 38.83% (TO35), and 39.13% (TO28) G + C content. Genome-based typing revealed that the CRAB strains belonged to ST2346 and ST231, while *Kp* strains belonged to different ST15, ST17, ST101, and ST512. Genomic characteristics of the MDR-GNB clinical isolates tested are shown in Table 1.

### 3.2. Bacterial Killing of DMSO Against Multidrug-Resistant Clinical Strains.

**Figure 1:**
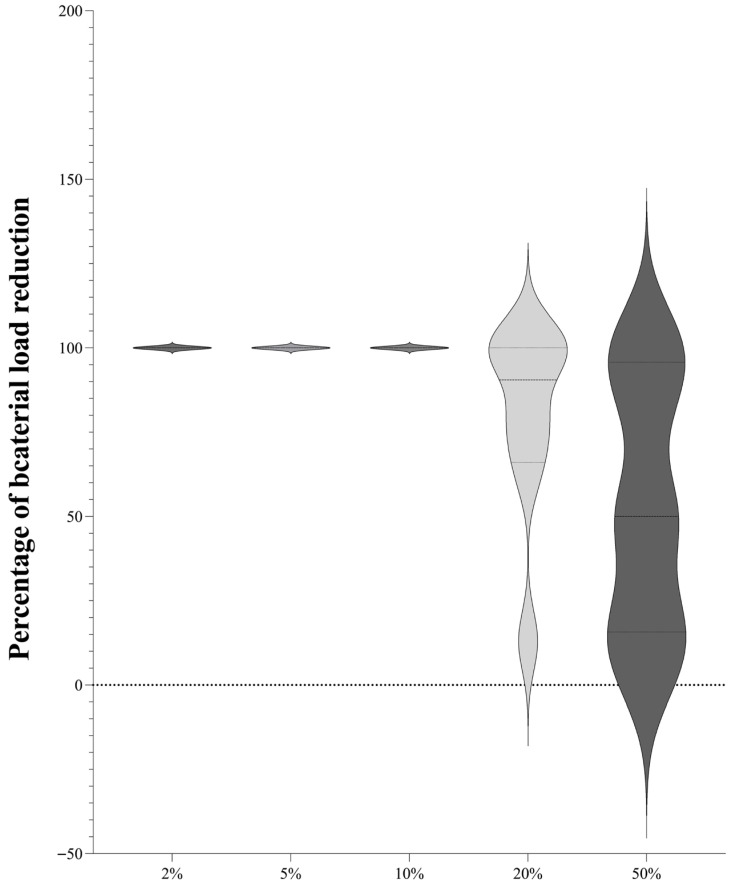
Percentage of bacterial load concentration in relation to control growth in the presence of escalating concentrations of dimethyl sulfoxide (DMSO), ranging from 2% to 50%.

To evaluate the potential antibacterial activity of DMSO against bacteria and to minimize the potential interference with synergy evaluation of cinnamaldehyde, eugenol, and carvacrol with antibiotics, different concentrations were tested (i.e., 2%, 5%, 10%, 20%, and 50% *v*/*v*), as shown in Figure 1.

Our results showed that DMSO at concentrations higher than 10% displayed a statistically significantly higher bacterial killing compared to control growth (Figure S1 in the Supplementary Material). Contrarily, at concentrations equal to or lower than 5%, DMSO had a modest effect on the exponential growth of bacterial cultures. Based on these findings, we selected DMSO at a concentration of 5% to resuspend the cinnamaldehyde, eugenol, and carvacrol powders, corresponding to a final concentration of 1% in the microtiter wells for MIC determination and synergy testing at high concentrations.

### 3.3. Antimicrobial Activity of Antibiotics, Eugenol, Carvacrol, and Cinnamaldehyde Against Multidrug-Resistant Clinical Strains

**Figure 2:**
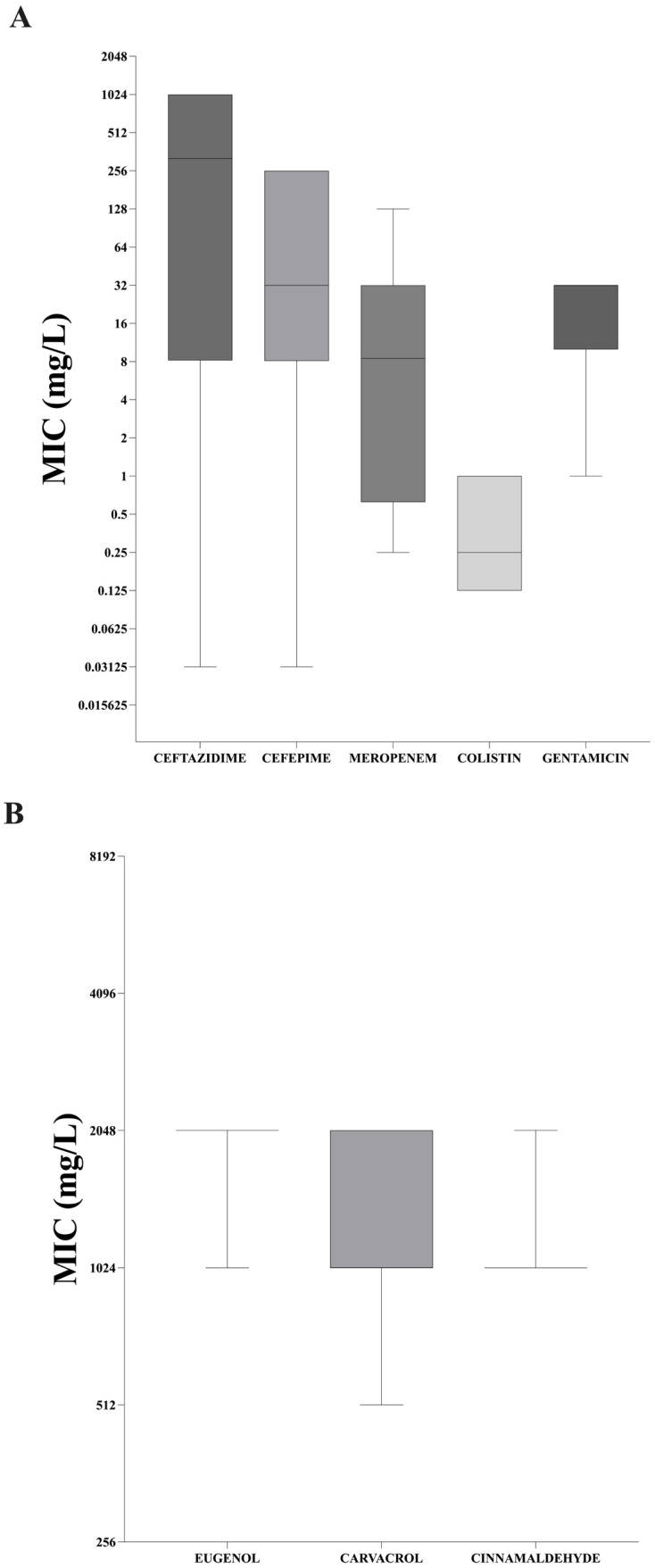
Antimicrobial activity of ceftazidime, cefepime, meropenem, colistin, or gentamicin (Panel (**A**)) and carvacrol, eugenol, and cinnamaldehyde (Panel (**B**)) against carbapenem-resistant *Acinetobacter baumannii* (CRAB), ESBL-producing, and carbapenemase-producing Enterobacterales (CPE) clinical strains.

In this study, a total of seven MDR-GNB were selected based on antimicrobial resistance characteristics. Antimicrobial susceptibility profiles of MDR-GNB clinical strains against different antimicrobials are shown in Figure 2A.

Phenotypic results showed that ceftazidime MICs ranged from 0.032 to 1024 mg/L (mean 468 mg/L; median 320 mg/L; IQR 8.188–1024 mg/L), cefepime exhibited MIC values between 0.032 and 256 mg/L (mean 108 mg/L; median 32 mg/L; IQR 8.094–256 mg/L), meropenem MIC values ranged between 0.25 and 128 mg/L (mean 26.34 mg/L; median 8.5 mg/L; IQR 0.625–32 mg/L). Colistin MICs also ranged from 0.125 to 1 mg/L (mean 0.48 mg/L; median 0.25 mg/L; IQR 0.125–1 mg/L), and gentamicin MIC values ranged between 1 and >32 mg/L (mean 23.13 mg/L; median 32 mg/L; IQR 10–32 mg/L). Against eugenol, MDR-GNB clinical strains tested in this study displayed MICs ranging from 1024 to 2048 mg/L (mean 1920 mg/L; median 2048 mg/L; IQR 2048 mg/L), while carvacrol exhibited MIC values from 512 to 2048 mg/L (mean 1344 mg/L; median 1024 mg/L; IQR 1024–2048 mg/L), while cinnamaldehyde MIC against MDR-GNB values ranged between 1024 and 2048 mg/L (mean 1152 mg/L; median 1024 mg/L; IQR 1024 mg/L). Antimicrobial susceptibility results against cinnamaldehyde, eugenol, and carvacrol against the MDR-GNB clinical strains included in this study are shown in Figure 2B.

### 3.4. Synergistic Activity of Eugenol in Combination with Antimicrobials Against Multidrug-Resistant Clinical Strains

**Figure 3:**
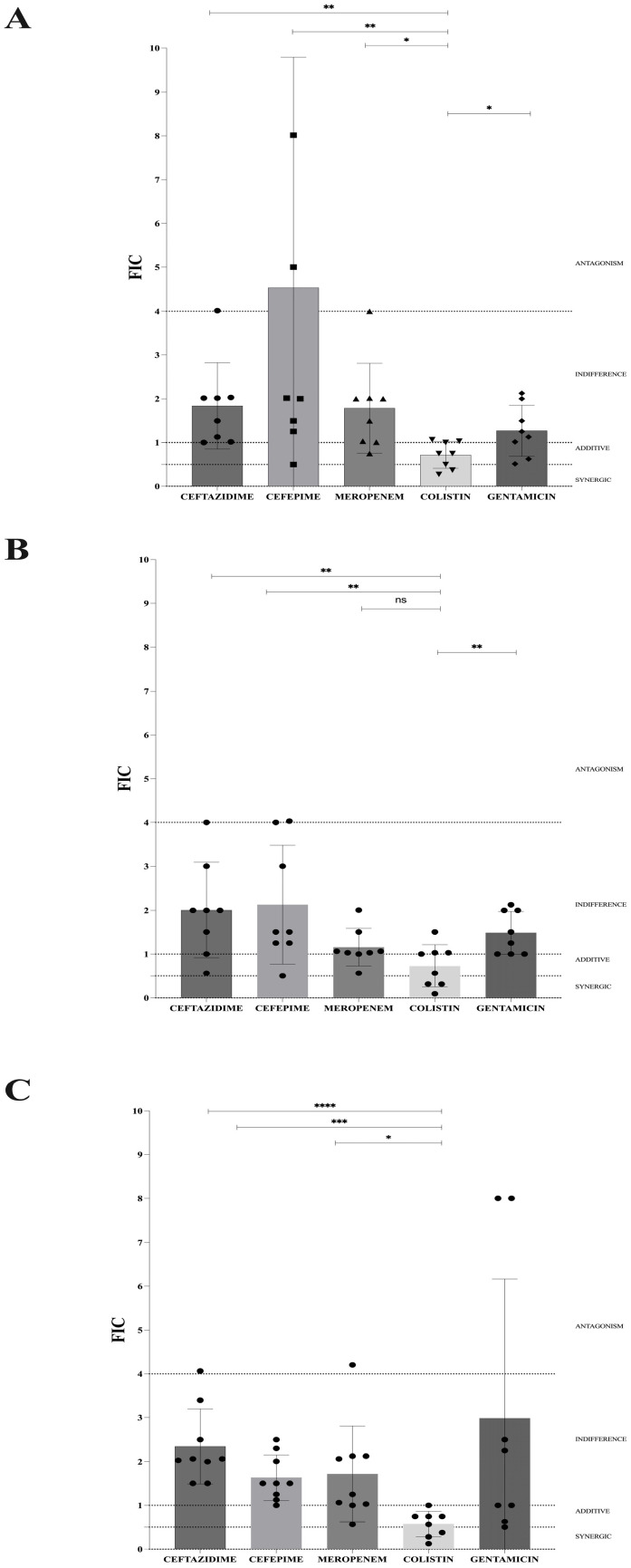
Fractional Inhibitory Concentration Index (FICI) of eugenol (Panel (**A**)), cinnamaldehyde (Panel (**B**)), and carvacrol (Panel (**C**)) in combination with β-lactams, gentamicin, or colistin against carbapenem-resistant *Acinetobacter baumannii* (CR*Ab*) ESBL-producing and carbapenemase-producing Enterobacterales (CPE) clinical strains. Statistically differences are highlighted with asterisks (* *p* < 0.05; ** *p* < 0.01; *** *p* < 0.001; **** *p* < 0.0001). ns: no significance.

Synergistic activity of eugenol in combination with different antimicrobials is shown in Figure 3A, showing that eugenol in combination with ceftazidime had indifferent effect against all strains, displaying additivity with meropenem (37.5%) and cefepime (12.5%), whereas none of these combinations had a synergistic effect. Of note, antagonism was observed with ceftazidime (12.5%), cefepime (25%), and meropenem (12.5%).

**Figure 4:**
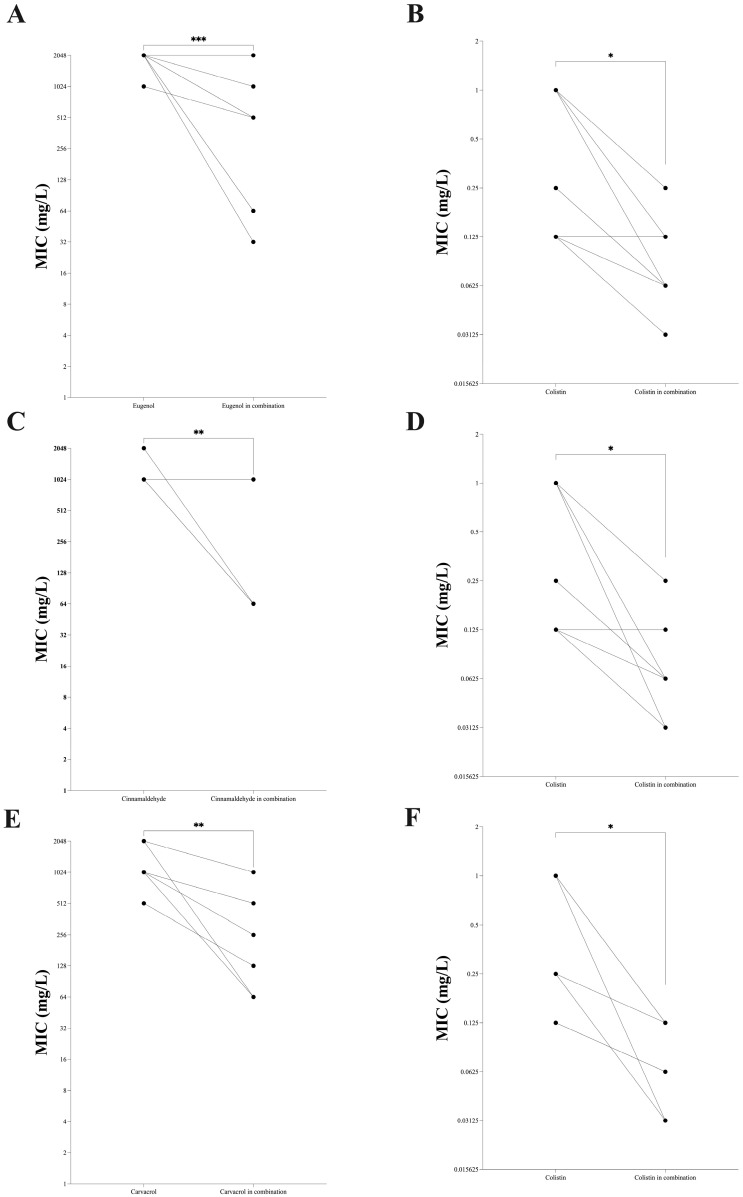
MIC reduction in essential oil molecules in the presence of colistin against multidrug-resistant (MDR) Gram-negative strains included in this study. Eugenol (Panel (**A**)) and colistin (Panel (**B**)) in association. Cinnamaldehyde (Panel (**C**)) and colistin (Panel (**D**)) in association. Carvacrol (Panel (**E**)) and colistin (Panel (**F**)) in association. Statistically differences (* *p* < 0.05; ** *p* < 0.01; *** *p* < 0.001).

When tested in combination with gentamicin, eugenol was indifferent in 37.5%, showed additivity in 62.5%, while no synergistic or antagonistic effect was observed against the MDR clinical strains included in this study. On the other hand, eugenol in combination with colistin displayed synergistic effect against 37.5%, additivity in 25%, and indifference in 37.5% of the MDR Gram-negative clinical isolates included in this study. Comparison between different combinations showed that eugenol in combination with colistin exhibited statistically significant FICI reduction compared with the combination of eugenol with β-lactams or gentamicin (Figure 3A; Figure S2A,B in the Supplementary Material). In detail, analysis of synergy testing showed that eugenol in combination with ceftazidime displayed FIC values from 1.002 to 4.016 (mean 1.840; median 1.758; IQR 1.043–2.027); with cefepime, the FICs ranged between 0.5 and 5 (mean: 2.783; median: 2008; IQR 1.313–7.262); in combination with meropenem, the FIC values were comprised between 0.75 and 4 (mean 1.788; median 1.750; IQR 1.014–2.012); with colistin, FIC values ranged from 0.281 to 1.063 (mean 0.7188; median 0.75; IQR 0.406–1.023), while in combination with gentamicin, eugenol showed FIC values between 0.516 and 2.125 (mean 1.270; median 1.188; IQR 0.722–1.875). Deeper examination of the synergy results showed that the combination of eugenol with colistin significantly reduced (*p* < 0.05) the MICs of both molecules when tested in association with the MICs of agents alone (Figure 4A,B). 

### 3.5. Synergistic Activity of Cinnamaldehyde in Combination with Antimicrobials Against MultiDrug-Resistant Clinical Strains

Cinnamaldehyde in combination with different β-lactams demonstrated indifference against 87.5% of the strains, whereas synergy was observed only against OXA-181 producers (Figure 3B). Similarly, cinnamaldehyde in combination with gentamicin exhibited an indifferent effect against all MDR-GNB strains. On the other hand, cinnamaldehyde in combination with colistin displayed a synergistic effect against 50% of clinical strains and indifference in 50% of MDR Gram-negative clinical isolates. In detail, cinnamaldehyde in combination with ceftazidime showed FIC values from 0.563 to 4 (mean 2.008; median 2; IQR 1.125–2.751), from 0.5 to 4.031 with cefepime (mean 2.129; median 1.5; IQR 1.25–3.75), from 0.563 to 2.008 with meropenem (mean 1.158; median 1.047; IQR 1.009–1.391), and from 0.094 to 1.5 with colistin (mean 0.7306; median 0.782; IQR 0.312–1.031), while FIC values ranged from 1.001 to 2.125 (mean 1.485; median 1.375; IQR 1.001–2) in combination with gentamicin. Synergistic activity of cinnamaldehyde in combination with different antimicrobials is shown in Figure 3B. In particular, cinnamaldehyde in combination with colistin revealed a statistically significant reduction in the FIC value compared with cinnamaldehyde combined with β-lactams or gentamicin (Figure 3B and Figure S2C,D in the Supplementary Material). Evaluation of synergy results showed that the combination of cinnamaldehyde with colistin was able to significantly decrease (*p* < 0.05) the MICs of both molecules against all clinical strains when tested in combination (Figure 4C,D).

### 3.6. Synergistic Activity of Carvacrol in Combination with Antimicrobials Against Multidrug-Resistant Clinical Strains

Carvacrol in combination with ceftazidime and cefepime showed indifference against all MDR strains, while in association with meropenem and gentamicin, it revealed an indifferent effect against 87.5% of clinical isolates, while synergistic activity was only observed against one isolate (Figure 3C). In detail, carvacrol showed FIC values from 1.5 to 4.063 mg/L (mean 2.215; median 2.047; IQR 1.751–2.95) with ceftazidime, from 1 to 2.5 mg/L (mean 1.548; median 1.5; IQR 1.188–2.154) with cefepime, and from 0.563 to 2.125 (mean 1.403; median 1.157; IQR 1.016–2.125) with meropenem, while FIC values ranged between 0.501 and 8.001 (mean 2.986; median 1.626; IQR 0.722–6.626) in combination with gentamicin (Figure 3C). Contrarily, carvacrol showed a synergistic effect against 50% of MDR strains and additivity against 37.5% of isolates included in this study when combined with colistin. In particular, carvacrol in combination with colistin displayed FIC values from 0.125 to 1 (mean 0.5743; median 0.6565; IQR 0.304–0.75), thus resulting in a statistically significant lower FIC value (*p* < 0.05 by Student’s *t*-test) compared to other carvacrol-based combinations (Figure 3C and Figure S2E,F in the Supplementary Material). Deeper analysis of the synergistic effect of the combination of carvacrol and colistin demonstrated a statistically significant reduction in the MICs of both compounds compared to when each was used individually (Figure 4E,F).

## 4. Discussion

Here, we evaluated the synergistic activity of eugenol, cinnamaldehyde, and carvacrol with different β-lactams, gentamicin, and colistin against representative MDR GNB clinical isolates. In particular, six clinical isolates exhibited the production of carbapenemase belonging to different classes (i.e., class A, B, and D carbapenemase following Ambler classification), while one isolate was an ESBL producer.

Our results demonstrated that eugenol, cinnamaldehyde, and carvacrol displayed synergistic activity in association with colistin against MDR-GNB clinical isolates including ESBL- and carbapenemase-producing strains. We also observed that the association between components of essential oils with β-lactams or gentamicin did not exert synergistic activity against most of the MDR-GNB clinical isolates, even if sporadic synergistic interactions were observed. Therefore, the potential clinical use of these combinations is unlikely to be clinically effective, as the synergy is insufficient to reduce MICs to clinically treatable levels. In contrast, combinations of eugenol, cinnamaldehyde, and carvacrol with colistin demonstrated potent in vitro activity against all MDR-GNB strains included in this study.

Our findings are consistent with those of previous investigations that demonstrated synergistic activity of molecules of essential oils combined with colistin against MDR-GNB [19,20,21]. In particular, colistin acts by targeting the outer membrane of GNB and, in particular, by binding to lipid A of lipopolysaccharides (LPSs), leading to bacterial membrane destabilization [22,23]. Carvacrol and eugenol cause disruption of bacterial membranes by altering membrane fluidity, increasing permeability, and causing leakage of intracellular contents [24,25,26]. To this end, the combination of colistin with these compounds may result in cumulative or complementary membrane damage, thus facilitating increased antibiotic uptake and enhancing bactericidal activity even against MDR-GNB strains [20,21]. Cinnamaldehyde has been shown to interfere with membrane-associated proteins and energy metabolism, further compromising bacterial homeostasis [25,26,27]. Based on these findings, we hypothesized that the combination of cinnamaldehyde with colistin may reduce the effective concentration of both molecules required for bacterial killing.

The rapid global spread of MDR-GNB represents one of the most critical challenges to modern antimicrobial therapy [28]. Resistance mechanisms such as β-lactamase production, target modification, reduced membrane permeability, and active efflux severely limit the efficacy of last-line antibiotics, including β-lactams, colistin, and aminoglycosides [29]. To this end, the identification of adjuvant molecules able to restore or enhance antibiotic activity is of particular importance, especially considering that their clinical application as standalone agents is limited by factors such as volatility, hydrophobicity, and relatively high MICs [30,31,32]. The observed synergistic interactions of eugenol, cinnamaldehyde, and carvacrol with colistin would hence suggest that these combinations may be more effective when used as antibiotic potentiators rather than as primary antimicrobial agents. Nevertheless, we acknowledge that this study has some limitations, including the relatively small number of strains analyzed, although representative of the principal carbapenemase-producing Gram-negative bacteria, which may not fully reflect clinical outcomes and the lack of analysis based on the bactericidal activity over time [32,33,34]. Consequently, these results need further investigation using a large number of clinical strains, evaluating the bacteria killing over time, and defining the bacterial load reduction.

## 5. Conclusions

In conclusion, the synergistic interactions between carvacrol, eugenol, and cinnamaldehyde with colistin represent a promising strategy to counteract MDR-GNB. This combination could be used to reduce the dosage of these molecules and colistin, potentially mitigating their toxicity to eukaryotic cells. We also hypothesized that the synergistic activities observed for these molecules in combination with colistin may be used for prophylaxis of MDR-GNB clinical strains in colonized patients, also considering their potential toxicity. Therefore, future studies should focus on the evaluation of the cytotoxic effect of this combination and its clinical applicability at different concentrations to fully exploit its potential in antimicrobial combination therapies.

## Figures and Tables

**Table 1:** Genetic characteristics of the multidrug-resistant (MDR) Gram-negative clinical strains included in this study.

Strain	Species	ST	Antimicrobial Resistance Determinants										Porins	
			β-Lactams		Macrolides	Aminoglycosides	Quinolones	Fosfomycin	Trimethoprim	Multidrug Efflux	Sulfonamides	Tetracyclines		
			Carbapenemase	B-Lactamase									*OmpK35*	*OmpK36*
LE08	*Klebsiella pneumoniae*	512	*bla*_KPC-31_	*bla*_TEM-1_ *bla*_SHV-11_	*mph(A)*	*aadA2*; *aac(6′)-Ib*	*gyrA_S83I*; *parC_S80I*	*fosA*	*dfrA12*	*emrD*; *oqxA*; *oqxB*	*-*	*-*	truncated at aa 41	Ins_GD-135
NA28	*Klebsiella pneumoniae*	101	*bla*_KPC-3_	*bla*_TEM-1_ *bla*_SHV-1_	*msr(E)*; *mph(E)*	*armA*	*gyrA_D87N*; *gyrA_S83Y*; *parC_S80I*; *qnrB1*	*fosA*	*dfrA14*	*emrD*; *oqxA*; *oqxB20*	*-*	*tet(A)*	WT	Ins_GD-135
Kp86	*Klebsiella pneumoniae*	17	*-*	*bla*_TEM-1B_ *bla*_SHV-172_ *bla*_CTX-M-15_	*-*	*aph(3″)-Ib*; *aph(6)-Id*; *aadA16*; *aac(3)-IIa*; *aac(6′)Ib-cr*	*qnrB6*; *OqxB*	*fosA6*	*dfrA27*	*-*	*sul2*	*-*	truncated at aa 41	truncated at aa 88
Kp61	*Klebsiella pneumoniae*	15	*bla*_NDM-1_	*bla*_OXA-10_ *bla*_TEM-1B_ *bla*_SHV-106_ *bla*_CMY-2_ *bla*_CTX-M-15_	*-*	*aph(3″)-Ib*; *aph(6)-Id*; *aadA1*; *aac(3)-IIa*; *aac(6′)-Ib3*	*OqxB*	*fosA6*	*dfrA14*	*-*	*sul2*	*-*	WT	WT
Kp51	*Klebsiella pneumoniae*	16	*bla*_OXA-181_	*bla*_SHV-199_	*-*	*-*	*qnrS1*; *OqxB*	*fosA5*	*-*	*-*	*-*	*-*	WT	truncated at aa 183
TO35	*Acinetobacter baumannii*	2346	*bla*_OXA-23_	*bla*_OXA-66_, *bla*_ADC-25_	*-*	*aph(3″)-Ib*; *aph(6)-Id*	*-*	*-*	*-*	*-*	*sul2*	*tet(B)*	NA	NA
TO28	*Acinetobacter baumannii*	231	*bla*_OXA-23_	*bla*_OXA-69_, *bla*_ADC-25_	*-*	*armA*; *aph(3″)-Ib*; *aph(6)-Id*; *aac(3)-Ia*; *ant(2″)-Ia*	*-*	*-*	*-*	*-*	*sul2*	*-*	NA	NA
BL21	*Escherichia coli*	NA	NA	NA	NA	NA	NA	NA	NA	NA	NA	NA	NA	NA

Abbreviations: NA, Not applicable; ST, Sequence Type; WT, Wild Type. Genetic analysis showed that all isolates carried antimicrobial resistance determinants to antimicrobials belonging to different classes. In detail, genes associated with resistance included aminoglycosides (*aadA2*; *aac(6′)-Ib*, *armA*, *aph(3″)-Ib*; *aph(6)-Id*; *aadA16*; *aac(3)-IIa*; *aac(6′)Ib-cr*, *ant(2″)-Ia*), fosfomycin (*fosA*), macrolides (*mph(A)*, *msr(E)*; *mph(E)*), trimethoprim (*dfrA*), quinolones (*gyrA*; *parC*; *qnrB6*; *OqxB*, *qnrS1*), sulfonamides (*sul1*; *sul2*), tetracyclines (*tet(A)*, *tet(B)*), as well as multiple genes involved in multi-drug efflux (*emrD*; *oqxA*; *oqxB*). Concomitantly, antimicrobial resistance to Β-lactams was found in 100% (5/5) of *Kp* harboring *bla*_TEM_ and *bla*_SHV_ genes and *bla*_ADC-25_ in 100% (2/2) of CR*Ab*. Four out of five *Kp* and all *Ab* carried carbapenemase genes (i.e., class A [*bla*_KPC_], B [*bla*_NDM_], and D [*bla*_OXA-23_] carbapenemase following Ambler’s classification). Sequence analysis of porin genes revealed that *OmpK35* had a sequence interruption at amino acid position 41 in two out of five *K. pneumoniae* clinical strains (Table 1). Sequence analysis of *OmpK36* porin resulted in a truncated form in two clinical strains, and a double insertion (aspartic acid and threonine) at amino acid position 135 in two out of five *K. pneumoniae*.

## Data Availability

The original contributions presented in this study are included in the article/Supplementary Material. Further inquiries can be directed to the corresponding author.

## Supplementary Materials

The following supporting information can be downloaded at https://www.mdpi.com/article/10.3390/antibiotics15040391/s1. Figure S1. Statistical comparison between bacterial load reduction in presence of dimethyl sulfoxide (DMSO) at concentrations of 10% vs. 20% (Panel A) and 10% vs. 50% (Panel B). Figure S2. Estimation plots of the Fractional Inhibitory Concentration Index (FICI) between eugenol in combination with ceftazidime vs. eugenol in combination with colistin (Panel A) and eugenol in combination with gentamicin vs. eugenol in combination with colistin (Panel B). Comparison between FICI of cinnamaldehyde in combination with ceftazidime vs. cinnamaldehyde in combination with colistin (Panel C) and cinnamaldehyde with gentamicin vs. cinnamaldehyde in combination with colistin (Panel D). Comparison between FICI of carvacrol in combination with ceftazidime vs. carvacrol in combination with colistin (Panel E) and carvacrol with gentamicin vs. carvacrol in combination with colistin (Panel F).
