# Supplementary material for: Synergistic Activity of Eugenol, Cinnamaldehyde, and Carvacrol in Combination with Different Antibacterial Agents Against Multidrug-Resistant Gram-Negative Clinical Isolates
Source: Antibiotics (Basel). 2026 Apr 11;15(4):391. doi: 10.3390/antibiotics15040391 (PMC13113955; doi:10.3390/antibiotics15040391)
Supplement: Supplementary file 1 [file antibiotics-15-00391-s001.zip › antibiotics-4227641-supplementary.pdf]

Figure S1. Statistical comparison between bacterial load reduction in presence of dimethyl sulfoxide (DMSO) at concentration of 10% vs 20% (Panel A) and 10% vs 50% (Panel B).

A

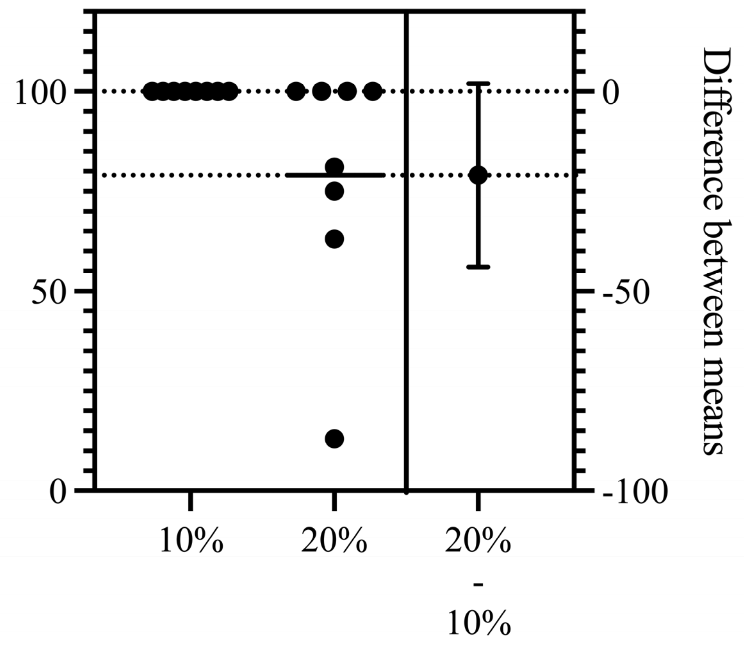

B

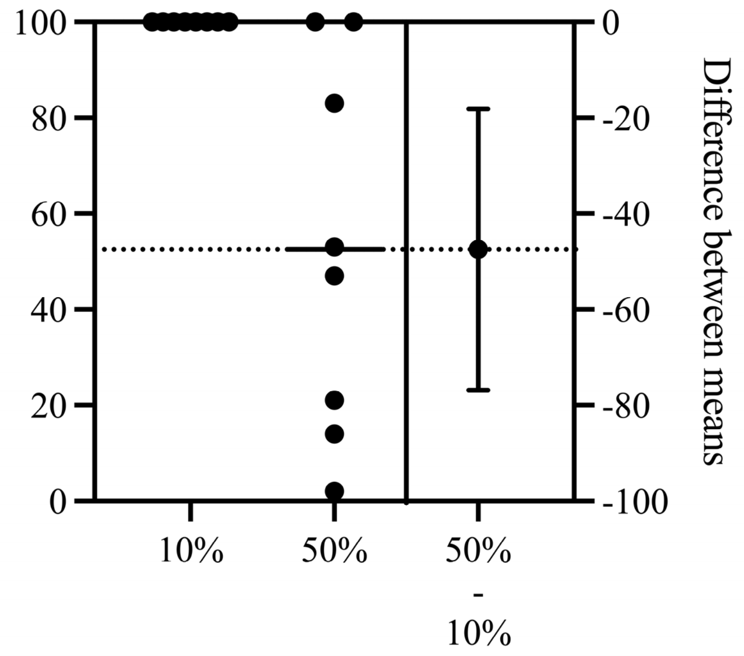

Figure S2. Estimation plots of the Fractional Inhibitory Concentration Index (FICI) between eugenol in combination with ceftazidime vs eugenol in combination with colistin (Panel A) and eugenol in combination with gentamicin vs eugenol in combination with colistin (Panel B). Comparison between FICI of cinnamaldehyde in combination with ceftazidime vs cinnamaldehyde in combination with colistin (Panel C) and cinnamaldehyde with gentamicin vs cinnamaldehyde in combination with colistin (Panel D). Comparison between FICI of carvacrol in combination with ceftazidime vs carvacrol in combination with colistin (Panel E) and carvacrol with gentamicin vs carvacrol in combination with colistin (Panel F).

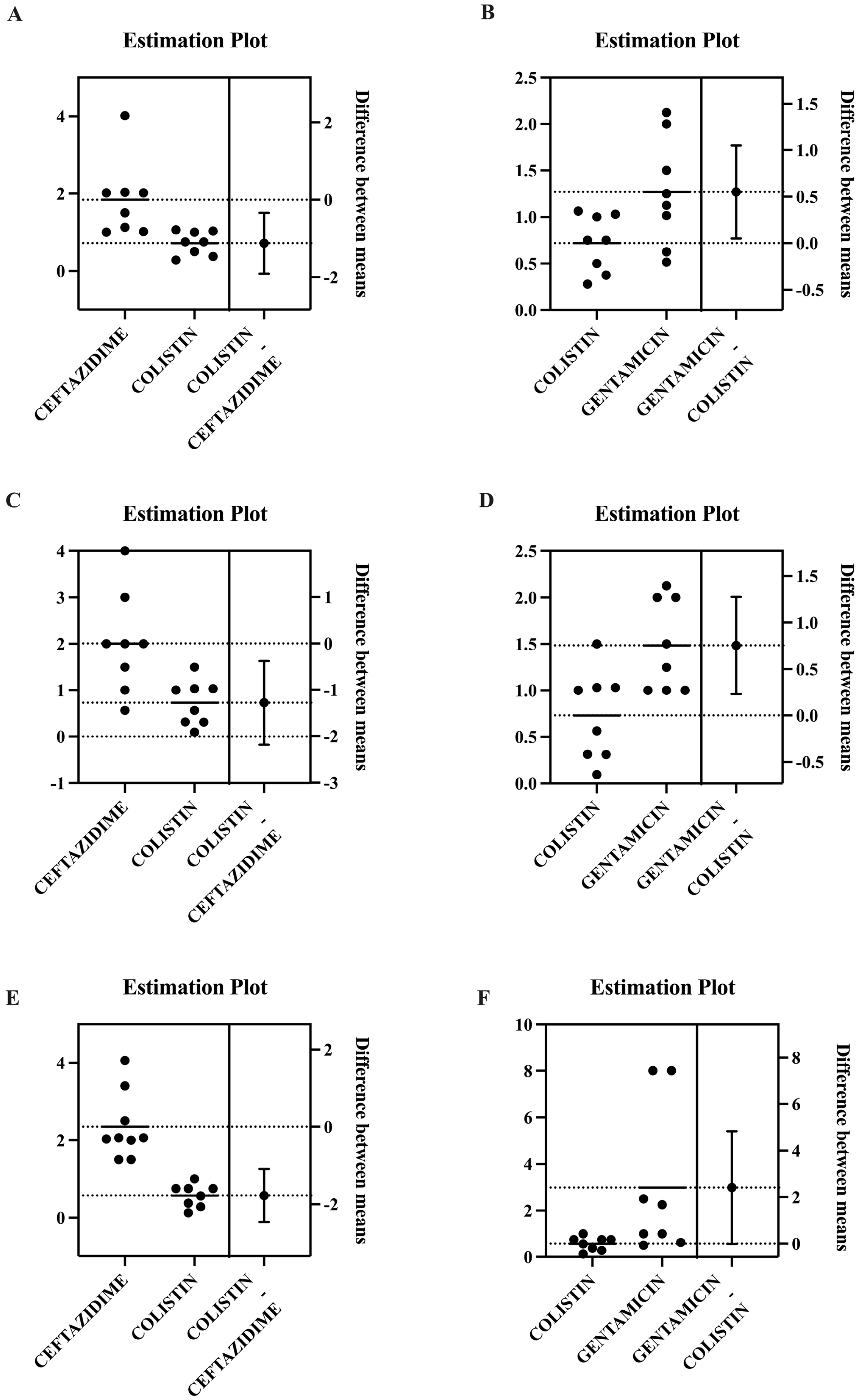
A
Estimation Plot
Difference between means
CEFTAZIDIME
COLISTIN
COLISTIN - CEFTAZIDIME
B
Estimation Plot
Difference between means
COLISTIN
GENTAMICIN
GENTAMICIN - COLISTIN
C
Estimation Plot
Difference between means
CEFTAZIDIME
COLISTIN
COLISTIN - CEFTAZIDIME
D
Estimation Plot
Difference between means
COLISTIN
GENTAMICIN
GENTAMICIN - COLISTIN
E
Estimation Plot
Difference between means
CEFTAZIDIME
COLISTIN
COLISTIN - CEFTAZIDIME
F
Estimation Plot
Difference between means
COLISTIN
GENTAMICIN
GENTAMICIN - COLISTIN
